# Male mice, caged in the International Space Station for 35 days, sire healthy offspring

**DOI:** 10.1038/s41598-019-50128-w

**Published:** 2019-09-24

**Authors:** Takafumi Matsumura, Taichi Noda, Masafumi Muratani, Risa Okada, Mutsumi Yamane, Ayako Isotani, Takashi Kudo, Satoru Takahashi, Masahito Ikawa

**Affiliations:** 1 Department of Experimental Genome Research, Research Institute for Microbial Diseases, Osaka University, 3-1 Yamada-oka, Suita, Osaka 565-0871 Japan; 2 Graduate School of Pharmaceutical Sciences, Osaka University, 1-6 Yamada-oka, Suita, Osaka 565-0871 Japan; 3 Department of Genome Biology, Faculty of Medicine, University of Tsukuba, 1-1-1 Tennodai, Tsukuba, Ibaraki 305-8575 Japan; 4 Mouse Epigenetics Project, ISS/Kibo experiment, Japan Aerospace Exploration Agency (JAXA), Tsukuba, Ibaraki Japan; 5 JEM Utilization Center, Human Spaceflight Technology Directorate, Japan Aerospace Exploration Agency (JAXA), Tsukuba Space Center, 2-1-1 Sengen, Tsukuba, Ibaraki 305-8505 Japan; 6 Laboratory Animal Resource Center in Transborder Medical Research Center, and Department of Anatomy and Embryology, Faculty of Medicine, University of Tsukuba, 1-1-1 Tennodai, Tsukuba, Ibaraki 305-8575 Japan; 7 Laboratory of Reproductive Systems Biology, Institute of Medical Science, The University of Tokyo, 4-6-1 Shirokanedai, Minato-ku, Tokyo, 108-8639 Japan; 8 Present Address: Laboratory of Biopharmaceutical and Regenerative Sciences, Institute of Molecular Medicine and Life Science, Yokohama City University Association of Medical Science, 3-9 Fukuura, Kanazawa-ku, Yokohama, Kanagawa 236-0004 Japan; 9 Present Address: Center for Animal Research and Education, Nagoya University, Furo-cho, Chikusa-ku, Nagoya, Aichi, 464-8601 Japan; 10 Present Address: Graduate School of Science and Technology, Nara Institute of Science and Technology, 8916-5 Takayama-cho, Ikoma, Nara 630-0192 Japan

**Keywords:** Reproductive biology, Environmental sciences

## Abstract

The effect on the reproductive system and fertility of living in a space environment remains unclear. Here, we caged 12 male mice under artificial gravity (≈1 gravity) (AG) or microgravity (MG) in the International Space Station (ISS) for 35 days, and characterized the male reproductive organs (testes, epididymides, and accessory glands) after their return to earth. Mice caged on earth during the 35 days served as a “ground” control (GC). Only a decrease in accessory gland weight was detected in AG and MG males; however, none of the reproductive organs showed any overt microscopic defects or changes in gene expression as determined by RNA-seq. The cauda epididymal spermatozoa from AG and MG mice could fertilize oocytes *in vitro* at comparable levels as GC males. When the fertilized eggs were transferred into pseudo-pregnant females, there was no significant difference in pups delivered (pups/transferred eggs) among GC, AG, and MG spermatozoa. In addition, the growth rates and fecundity of the obtained pups were comparable among all groups. We conclude that short-term stays in outer space do not cause overt defects in the physiological function of male reproductive organs, sperm function, and offspring viability.

## Introduction

Since the first human flight into outer space by Gagarin in 1961^1^, more than 550 astronauts have traveled to outer space^2^. Recently, private enterprises have begun to prepare space travel services at a low price. The era where people can easily go into space is coming. However, there are many issues to be cleared in advance, such as launch or landing stress, psychological stress, gravity changes, and radiation. Specifically, gravity at launch and landing is up to 7.0 gravity^3^. Hypergravity leads to a decrease of cerebral blood flow and arterial pressure^4^. Zero gravity causes a deterioration of an astronaut’s muscles and bones^5,6^ and a disorder of the optic nerve^7^. The radiation dose per day on the International Space Station (ISS) is comparable to about half a year on Earth^8,9^, resulting in genomic mutations and cancer formation^10^. Thus, these factors have negative effects on the physiological functions of the human body.

Studies of the effects of space environment on the reproductive system are necessary to prevent undesirable effects in the next generation. Medaka^11^, sea urchins^12^, *Pleurodeles waltl*^13^, *Xenopus laevis*^14,15^ and birds^16^ caged in outer space did not show any overt defects on their reproductive ability. Wakayama *et al*. showed that freeze-dried mouse spermatozoa stored on the international space station (ISS) for 9 months are damaged by radiation^17^. Abnormal spermatogenesis and a decrease in sperm counts were observed in rats caged in outer space for 13 days^18,19^. However, the effects of space environment on the male reproductive organs at the molecular level remain unknown due to a limited sample size. Further, the effect of space environment on sperm fertilizing ability, male accessory glands, and progeny is also unclear.

To address those concerns, our project team has developed the Transportation Cage Unit (TCU) and Habitat Cage Unit (HCU)^20,21^. The HCU is capable of being installed in the Centrifuge-equipped Biological Experiment Facility (CBEF) on the ISS. This platform allowed us to cage mice under artificial gravity (AG) by centrifugation. In this mission, 12 male mice were caged individually under AG (≈1 gravity) and MG (≈μg) at The Japanese Experimental Module on the ISS for 35 days, and then transported back safely from space. The living mice in outer space are subjected to some effects of space travel (such as radiation, stress from landing and launching), but we can specifically evaluate the effect of changes in gravity by comparing between the AG and MG mice. Here, we discovered that the physiological function of male reproductive organs at the molecular level and sperm fertilizing ability of mice that return from the ISS were normal, and that the next generation (F1 generation) did not show any overt defects in growth rates and fecundity.

## Results

### Effect of space environment on male reproductive organs

**Figure 1:**
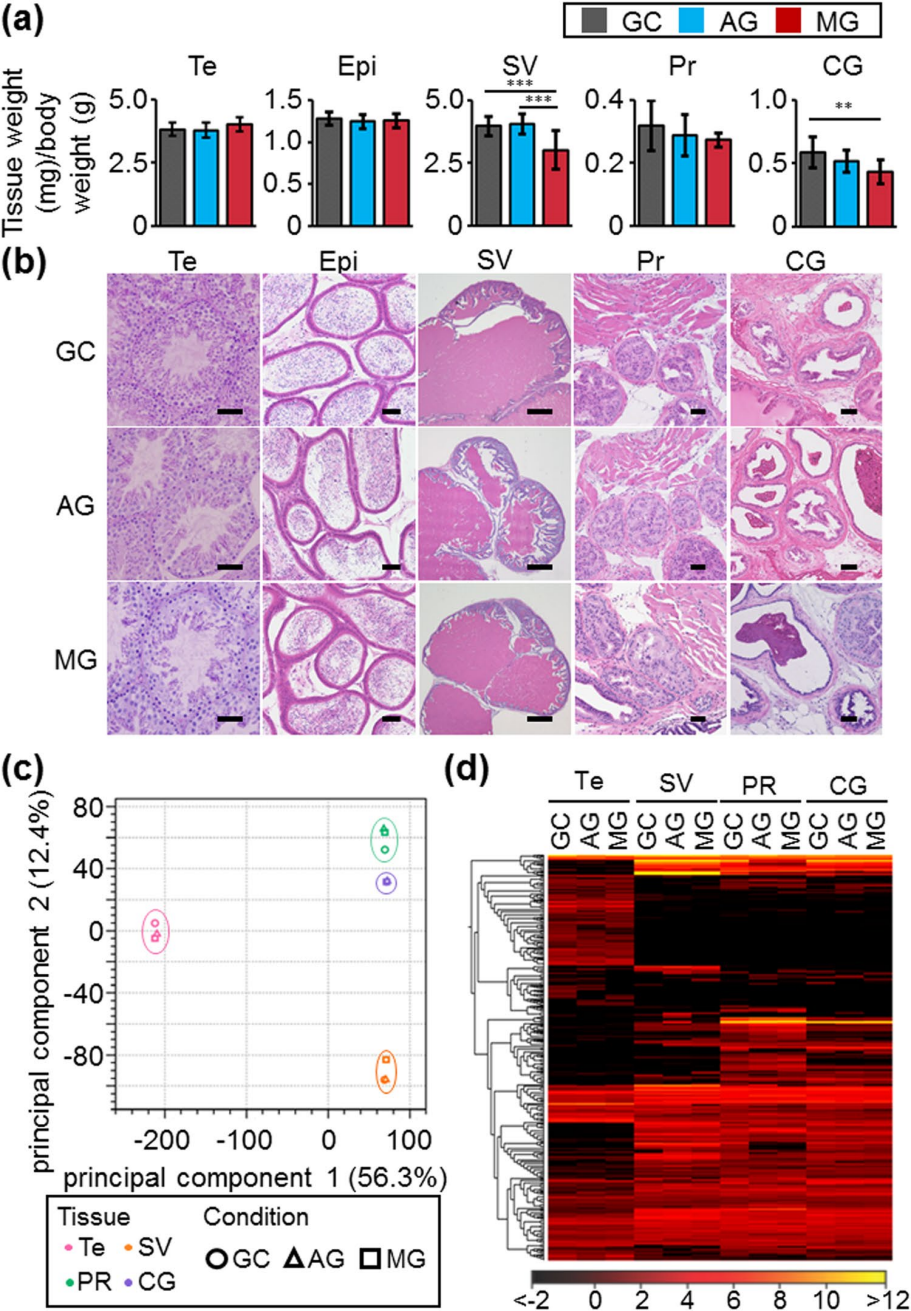
Characterization of male reproductive organs from AG and MG males. (**a**) Tissue weights. Each male reproductive organ was collected from 6 males per experimental group. Te: Testis, Epi: Epididymis, SV: Seminal vesicle, Pr: Prostate, CG: Coagulating gland. Data are the mean ± standard deviation. **P < 0.01, ***P<0.001. (**b**) Histological analysis. Plastic sections of the testis and other organs were stained by PAS-Hematoxylin and H&E, respectively. Scale bars show 50 µm (testis, epididymis, prostate, coagulating gland), and 500 µm (seminal vesicle). (**c**) Principal component analysis of RNA-seq data. The mixture of total RNA from 3 males in each experimental group was used for RNA-seq analysis. (**d**) Hierarchical clustering of genes with more than 2-fold change.

To reveal the influence of space environment on male reproductive organs, we characterized testes, epididymides, seminal vesicles, prostates, and coagulating glands collected from mice caged on the ISS for 35 days. Our project team developed the hardware unit that generates artificial gravity (≈1 gravity) (AG) in outer space by centrifugal force^20,21^. The hardware unit enables us to examine the influence of space environment on male fecundity by comparing AG and MG mice. In this study, we used not only the ground-control (GC) but AG mice as controls. There were no differences in the weights of testes and epididymides among GC, AG, and MG males [testis weight (mg)/body weight (BW) (g): 3.82 ± 0.25 (GC), 3.79 ± 0.29 (AG), 4.01 ± 0.29 (MG), epididymis weight (mg)/BW (g): 1.28 ± 0.08 (GC), 1.25 ± 0.08 (AG), 1.25 ± 0.08 (MG)] (Fig. 1a). Those of the seminal vesicles in MG males were significantly reduced, compared to GC and AG males [seminal vesicle weight (mg)/BW (g): 3.97 ± 0.40 (GC), 4.06 ± 0.40 (AG), 3.02 ± 0.77 (MG)] (Fig. 1a). The weights of prostates and coagulating glands in AG and MG males were lower than the GC males [prostate weight (mg)/BW (g): 0.32 ± 0.08 (GC), 0.29 ± 0.07 (AG), 0.27 ± 0.02 (MG), coagulating gland weight (mg)/BW (g): 0.59 ± 0.12 (GC), 0.52 ± 0.09 (AG), 0.43 ± 0.09 (MG)] (Fig. 1a). Meanwhile, AG and MG males did not show any defects in the histology of the testes, cauda epididymides, and accessary glands (Fig. 1b). Principal component analysis and profiling of RNA-seq data showed that gene expression levels in AG and MG males was comparable to GC males in all male reproductive organs (Fig. 1c,d). These results indicate that accessary gland weight is susceptible to a space environment, but histological features and gene expression of male reproductive organs in mice housed in space are normal.

### Sperm fertilizing ability of space mice *in vitro*

**Figure 2:**
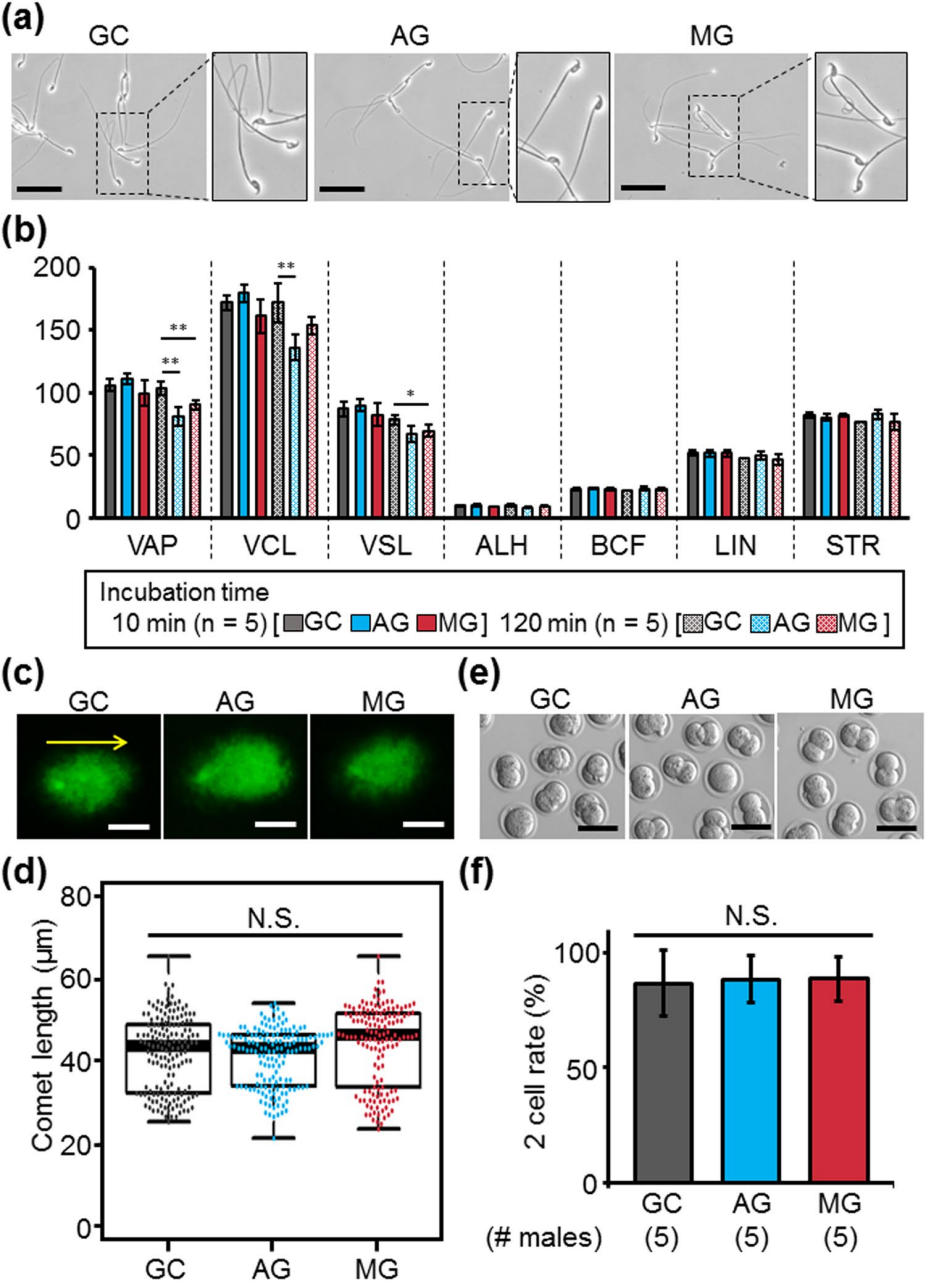
Characterization of cauda epididymal spermatozoa from AG and MG males. (**a**) Sperm morphology. Scale bars are 50 µm. (**b**) Sperm motility parameters. VAP: average path velocity, VCL: curvilinear velocity, VSL: straight line velocity, ALH: amplitude of lateral head displacement, BCF: beat cross frequency, LIN: linearity, and STR: straightness. Data are the mean ± standard deviation. *p < 0.05, **p < 0.01. (**c**) Comet assay images. The yellow arrow and scale bars show the comet length and 10 µm, respectively. (**d**) Comet length. Each plot indicates a comet tail from spermatozoon. Bounds of the box spans from 25 to 75% percentile, center line represents median, and whiskers visualize all data points. N.S.: not significant. (**e**) Fertilized eggs obtained by *in vitro* fertilization (IVF). After 24 hours of IVF, embryos were observed. Scale bars are 100 µm. (**f**) Fertilization rates by IVF. Data are the mean ± standard deviation. N.S.: not significant.

To reveal the influence of space environment on male fecundity, we characterized cauda epididymal spermatozoa from mice housed in space. Cauda epididymal sperm count from AG and GC males was comparable to GC males (Fig. 1b). We could not see any defects in sperm morphology among GC, AG, and MG males (Fig. 2a), but some parameters of sperm motility from AG and MG mice decreased 2 hours after incubation (Fig. 2b). The lengths of DNA in the comet tails of AG and MG spermatozoa were comparable to the GC spermatozoa (GC: 41.7 ± 9.1 µm, AG: 41.0 ± 7.1 µm, MG: 43.4 ± 9.6 µm) (Fig. 2c,d). These results indicate that space environment including radiation has minimal damage on spermatozoa *in vivo*. The AG and MG spermatozoa could efficiently fertilize oocytes at a comparable level as GC spermatozoa (GC: 86.7 ± 14.3%, AG: 88.5 ± 10.3%, MG: 88.6 ± 9.6%) (Fig. 2e,f). Thus, we revealed that spermatozoa from males caged in outer space have normal fertilizing ability.

### Embryogenesis of fertilized eggs, and fecundity of the next generation

**Figure 3:**
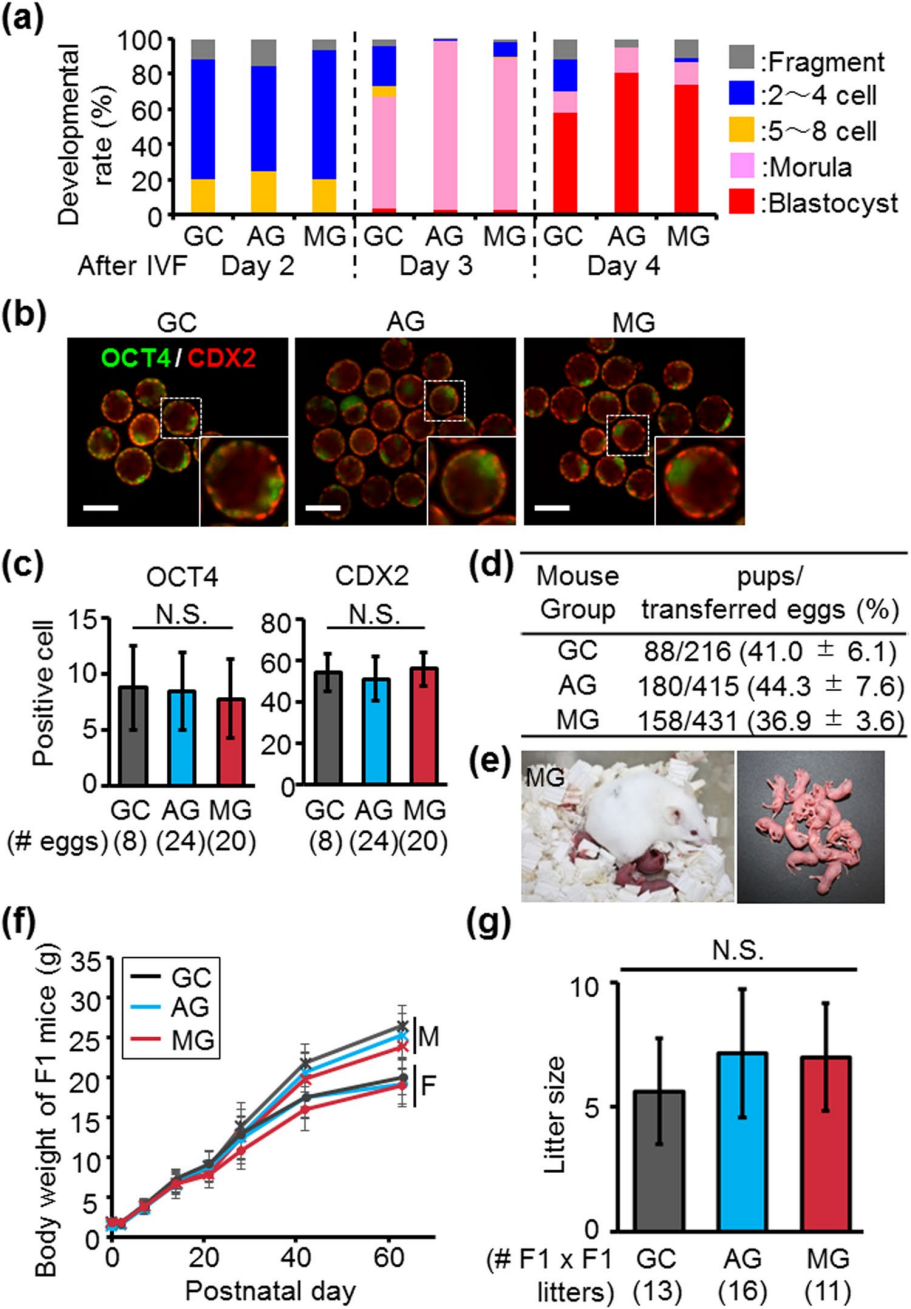
Characterization of fertilized eggs and F1 mice from AG and MG males. (**a**) Embryogenesis of fertilized eggs. Embryogenesis was observed for 4 days after IVF. (**b**) Immunostaining of OCT4 and CDX2 in blastocysts. OCT4 and CDX2 were used as markers for ICM and TE cells in blastocysts, respectively. Scale bars are 100 µm. **(c)** Quantification of OCT4- and CDX2-positive cells in blastocysts. Data are the mean ± standard deviation. N.S.: not significant. (**d**) Rates of delivered pups. Fertilized eggs obtained by IVF were transferred into pseudopregnent females. (**e**) Offspring from fertilized eggs with MG spermatozoa. (**f**) Growth curve of F1 mice. Body weight was monitored by postnatal day 63. “M” and “F” show the male and female mice, respectively. (**g**) Litter sizes of F1 × F1 intercrosses. Data are the mean ± standard deviation. N.S.: not significant.

To further analyze the developmental ability of eggs fertilized with spermatozoa from mice living in space, we observed embryogenesis for 4 days after *in vitro* fertilization. The developmental speed of fertilized eggs with AG and MG spermatozoa was comparable to that with GC spermatozoa, and these embryos efficiently developed to blastocysts (GC: 58.3%, AG: 80.9%, MG: 73.5%) (Fig. 3a). Wakayama *et al*. showed that ICSI embryos with freeze-dried mouse spermatozoa preserved in the ISS have decreased cell numbers in the trophectoderm (TE)^17^. Meanwhile, *in vitro* fertilized eggs with AG and MG spermatozoa did not show decreased cell numbers in either the inner cell mass (ICM) or TE [ICM (OCT4 was used as the ICM marker): 8.75 ± 3.77 (GC), 8.46 ± 3.44 (AG), 7.75 ± 3.51 (MG), TE (CDX2 was used as the TE marker): 54.38 ± 9.05 (GC), 51.42 ± 10.48 (AG), 56.00 ± 8.23 (MG)] (Fig. 3b,c). When we transferred these embryos into a pseudo-pregnant female, we obtained pups (F1 generation) from fertilized eggs with AG and MG spermatozoa at a comparable level to GC (GC: 41.0 ± 6.1%, AG: 44.3 ± 7.6%, MG: 36.9 ± 3.6%) (Fig. 3d). These offspring looked healthy (Fig. 3e), and their growth curves were normal (Fig. 3f). These results indicate that quality of fertilized eggs with spermatozoa from space males is normal. We did not see any significant differences among offspring obtained from F1 × F1 intercrosses (GC: 5.56 ± 3.04, AG: 7.15 ± 2.58, MG: 6.81 ± 2.27) (Fig. 3g). This result demonstrates that the effects of space environment on the F0 generation are not carried over to the fecundity of future generations.

## Discussion

There are some reports on the effects of space environment (*e.g*., microgravity, stress due to launching and landing, and radiation) on living mice. Specifically, the female and/or male mice that were caged for up to 90 days in the missions of The National Aeronautics and Space Administration (NASA) (https://www.nasa.gov/ames/rodent-research)^22^. The Russian Bion-M 1 biosatellite accommodated male mice in groups for 30 days^23,24^. The Italian Mice Drawer System housed 6 male mice individually for 91 days on the ISS^25–27^. However, in the latter two experiments, more than 50% of male mice died due to various reasons, such as health status and payload-related reasons^23–27^. Thus, the effect of space environment on a living body remains unclear due to a limited sample size. To overcome this issue, our project team newly developed two mouse habitat cages (TCU and HCU)^20,21^, that made it possible to house the mice under artificial gravity by centrifugation. In this mission, our project team caged individual male mice under AG (≈1G) and MG (≈μg) for 35 days on the ISS, and then all the mice were returned to Earth, indicating that we can purely compare the effect of gravity on the living body between AG and MG mice. Here, we analyzed the effect of space environment on the male reproductive system and sperm fertilizing ability at the molecular level.

The weights of the male accessory gland (seminal vesicles, prostates, and coagulating glands) decreased in MG males (Fig. 1a). It should be noted that the accessory gland weight slightly decreased in AG males (Fig. 1a). Meanwhile, the weights of the testes and epididymides did not decrease in either group. It is well-known that the majority of accessory gland weight is composed of secretions^28,29^. A previous report showed a decrease in plasma volume and total blood volume during spaceflight^5^. Thus, our results suggest that the weight of those organs which store secretions is also susceptible to space environment (gravity and other factors). Previous data showed that the accessory gland weight in castrated male mice is lower than controls due to a decrease in serum testosterone concentrations^30^. The decrease of testosterone levels has been observed during flight and postflight in rats and humans^31,32^. It would be interesting to examine the effect of space environment on male reproductive hormone levels, and whether changes of testosterone levels may lead to the decrease in accessory gland weight.

Previous work examined the amount of cytoskeletal proteins, sperm-specific proteins, and epigenetic event-related proteins (DNA-methylases, DNA demethylases, DNA acetylases, and histone deacetylases) in testes and vas deferens of mice exposed to space flight conditions for 21–24 days^33^. In fact, they showed that the expression levels of several genes in testes [Beta-tubulin, DNA methyltransferase (cytosine-5) 1, histone aminotransferase 1, histone deacetylase 1, lysine (K)-specific demethylase 5B, and tet methylcytosine dioxygenase 2] changed with qPCR, but that the quantitative change in these proteins was not detected. Meanwhile, we could not detect the change of the expression level of these genes with RNA-seq. The differences in some conditions (such as living environment, period for space flight) may lead to the discrepancy between the previous paper and our study.

The previous report showed that freeze-dried spermatozoa stored in the ISS for 9 months have DNA damage, and that the cell number in TE in embryos generated by these spermatozoa decreased^17^. However, we could not observe these defects in spermatozoa and embryos with AG and MG males caged in ISS for 35 days (Figs 2c,d, 3b,c). The discrepancy between the previous report and this study may be caused by the length of exposure to the radiation in the ISS or the repair of DNA damage in the individuals, leading to the more severe phenotypes in freeze-dried spermatozoa. We revealed that the growth rates and fecundity of the F1 generation from AG and MG males were normal (Fig. 3f,g). As the previous studies indicated that MG induced epigenetic changes in human lymphocytes^34^ and human twins (https://www.nasa.gov/feature/nasa-twins-study-investigators-to-release-integrated-paper-in-2018)^35^, in the future we need to analyze the effects of space environment on the epigenetics of male reproductive organs in mice caged in the ISS and the next generation.

Here, we revealed that the function of male reproductive organs at the molecular level and sperm fertilizing ability were normal in F0 mice caged in the ISS with AG and MG for 35 days. Further, the F1 generation from AG and MG males did not show any overt defects in growth rates and fecundity. In mice, spermatogenesis in testis and sperm maturation in the epididymis take 35 days and 10 days, respectively^36,37^. The 35-days stay in the ISS covered spermatogenesis and sperm maturation in outer space, respectively. Further analyses are required to examine the long-term effects of space environment on the male reproductive system. However, our results indicate that a short stay in space does not cause a decrease in male fertilizing ability and an undesirable effect in the next generation.

## Methods

### Animals

Five-week-old C57BL/6J male mice (Stock #000664) were purchased from The Jackson Laboratory (USA) for the space experiments and from Charles River Laboratories (Japan) for ground experiments. These mice were used for the experiments as described previously^20^. In brief, 12 male mice caged in the TCU were launched aboard the SpaceX Falcon 9 rocket (SpX9) on 18 June 2016. The Dragon space vehicle from SpX9 reached the ISS on June 20, and then mice were transferred to the HCU by the crew. We divided the mice into 2 groups of six mice each [AG (≈1G, at a centrifugation speed of 77 rpm) and MG], and they were caged in the ISS for about 35 days. This time span was decided by the flight schedule of this mission [mission #: Mouse habitat Unit-1 (MHU-1)]. All the mice returned to the Pacific Ocean offshore from California on 26 August 2016, and then they were sacrificed at the laboratory 2 days after splash down. The radiation load during the space experiment was measured with a ‘Bio Passive Dosimeter for Life Science Experiments in Space’ (Bio PADLES) (0.23 ± 0.02 mGy/d for the absorbed dose rate, and 0.43 ± 0.03 mSv/d for the dose equivalent rate). For replicating the housing conditions of the space flight experiment, we caged 6 males for 35 days at the JAXA Tsukuba Space Center in Japan.

Eight-week-old C57BL/6J female mice were purchased from SLC (Japan) for *in vitro* fertilization. ICR pseudopregnant female mice were purchased from SLC for embryo transfer. All animal experiments were conducted in accordance with the guidelines of “Animal experiment rules” established by the Research Institute for Microbial Diseases, Osaka University, and were approved by the Animal Care and Use Committee of the Research Institute for Microbial Diseases, Osaka University (#Biken-AP-H30-01).

### Body weight and sample collection

Body weight and sample collection were performed as described previously^20^. Testes, epididymides, coagulating glands (also known as the anterior prostate), prostates (mixture of dorsal, lateral, and ventral regions), and seminal vesicles were used for this study. The production of frozen spermatozoa was performed as described previously^38^. Specifically, R18S3 medium (ARK Resource) and PB1 (ARK Resource) were used to freeze spermatozoa.

### Histology

Testes, epididymides, seminal vesicles, prostates, and coagulating glands were fixed in 4% paraformaldehyde (Wako) for 1 day. After fixation, these samples were placed in PBS for 5 days, and then subjected to plastic embedding using Technovit® 8100 (Mitsui chemicals). For histological analysis of testes, 5 µm sections were stained with 1% periodic acid solution (Wako) for 10 minutes, followed by treatment with Schiff’s reagent (Wako) for 20 minutes, and then Mayer’s hematoxylin solution (Wako) for 5 minutes. For histological analysis of other tissues, 5 µm sections were stained in Mayer’s hematoxylin solution for 3 minutes, followed by treatment with eosin solution [53% (v/v) ethanol, 0.3% (v/v) eosin, and 0.5% (v/v) acetic acid] for 3 minutes. After dehydration with ethanol, these slides were mounted with Entellan® new (Merck Millipore) and observed under a BZ-X710 microscope (Keyence).

### Gene expression analysis

Total RNA from testes, coagulating glands, prostates, and seminal vesicles were obtained using TRIZOL® reagent (Thermo Fisher Scientific). RNA quality was examined using an RNA 6000 Pico kit (Agilent). Total RNA from 3 males in each experimental group was mixed. Total RNAs (50 ng) were used for RNA-seq library preparation using the NEBNext® rRNA Depletion Kit (New England Biolabs) and NEBNext® Ultra Directional RNA Library Prep Kit (New England Biolabs), and then 2 × 36 base paired-end sequencing was performed with NextSeq500 (Illumina) by Tsukuba i-Laboratory LLP (Tsukuba). FASTQ files were analyzed using CLC Genomics Workbench (Version 7.5.1; Qiagen). Sequences were mapped to the mouse genome (mm10) and quantified for annotated genes. Transcription expression values were estimated as “reads per kilobase per million reads”.

### Sperm motility and morphology

The frozen-thawed spermatozoa were incubated in TYH medium^39^. After 10 minutes and 2 hours of incubation, the sperm motility was recorded using an Olympus CX41 microscope equipped with a MiniTherm stage warmer (Sony) and a CM-040 GE CCD camera (JAI A/S) for 0.75 seconds at 37 °C. Spermatozoa were analyzed using the default program (Mouse CytoD 4x dark field settings) of the CEROS II sperm analysis system (software version 1.5.2; Hamilton Thorne Biosciences). More than 200 spermatozoa were analyzed in each sample. The morphology of the remaining spermatozoa was observed under a BX50F phase contrast microscopy (Olympus).

### Sperm comet assay

The sperm comet assay was done using the CometAssay® Kit (Trevigen). Specifically, the frozen-thawed spermatozoa were incubated in TYH medium for 30 minutes at 37 °C in an incubator with 5% CO_2_. The spermatozoa were diluted with 50 µl PBS (final conc.: 1 × 10^5^/ml), mixed with molten low-melting-point-agarose (Trevigen), and then put on the comet slide. Sample slides were immersed in lysis solution (Trevigen) with 40 mM dithiothreitol at 4 °C for 20 minutes, and then Actinase (final conc.: 10 µg/ml) was added. After incubation for 2 hours at room temperature, the slides were immersed in alkaline solution for an hour at 4 °C. After electrophoresis for 40 minutes at 1 Volt/cm, the slides were stained by SYBR Gold (Thermo Fisher Scientific). The comet tail length of about 30 spermatozoa per slide was measured with ImageJ (NIH).

### *In vitro* fertilization and embryogenesis

Pregnant mare serum gonadotropin (PMSG) (5 units, ASKA Pharmaceutical) was injected into the abdominal cavity of C57BL/6J female mice, followed by human chorionic gonadotropin (hCG) (5 units, ASKA Pharmaceutical) 48 hours after PMSG. After 14 hours of hCG injection, the oocytes were collected from the ampulla of each oviduct, and then incubated in CARD MEDIUM (KYUDO). Frozen-thawed spermatozoa were incubated in FERTIUP medium (KYUDO) for 30 minutes. The sperm suspension was added to CARD MEDIUM. After 3 hours of insemination, the eggs were moved to KSOM medium, and then incubated for 4 days at 37 °C in an incubator with 5% CO_2_. Each day after IVF, the embryos were observed under a phase contrast microscopy (Olympus).

### Embryo transfer

Two-cell stage embryos were transferred to the ampulla of pseudo-pregnant ICR mice. After 19 days of embryo transfer, offspring were obtained by caesarean section and natural childbirth.

### Immunostaining of blastocysts

After 4 days of IVF, the quality of blastocysts was examined as described previously^40^. Specifically, rabbit anti-mouse OCT3/4 polyclonal antibody (a marker for ICM cells, 1:200, MBL Code No. PM048) and mouse anti-CDX2 monoclonal antibody (a maker for TE cells, 1:150, MBL Code No. B-MU392AUC) were used as the primary antibodies. The secondary antibodies were Alexa Fluor 488-labeled goat anti-rabbit IgG (Thermo Fisher Scientific) or Alexa Fluor 546-labeled goat anti-mouse IgG (Thermo Fisher Scientific). The 70 images of embryos were captured at 1.2 µm intervals along the z-axis using a BZ-X710 microscope (Keyence). The cell number of ICM and TE in Z-stack images was counted.

### Body weight and fecundity of F1 generation

The body weight of the F1 generation was recorded at postnatal days 0, 3, 7, 14, 21, 28, 42, and 56. F1 mice were housed together with their foster mothers up to 4 weeks after birth. At 10 weeks of age, individual males were caged with 2 females for 2 months.

### Statistical analysis

All values are shown as the mean ± SD of at least three independent experiments. Statistical analyses were performed using Dunnett T3 method (for Figs 1a, 2b,d) and Tukey method (for Figs 1a, 2f, 3c,d,g), after examining the normal distribution and variance. Significance was defined as p-value < 0.05 using the following notations: *p < 0.05, **p < 0.01, and ***p < 0.001.

## References

[1] West JB (2001). Historical aspects of the early Soviet/Russian manned space program. J Appl Physiol (1985).

[2] Kerschmann RL (2018). The Challenge of Human Pathobiology in Space. Current Pathobiology Reports.

[3] Akiyama, T. The Pleasure of Spaceflight *The Journal of Space Technology and Science* **9**, 21–23 (1993).

[4] Konishi T (2018). Time-Dependent Changes in Cerebral Blood Flow and Arterial Pressure During Mild +Gz Hypergravity. Aerosp Med Hum Perform.

[5] Williams D, Kuipers A, Mukai C, Thirsk R (2009). Acclimation during space flight: effects on human physiology. CMAJ.

[6] Keyak JH, Koyama AK, LeBlanc A, Lu Y, Lang TF (2009). Reduction in proximal femoral strength due to long-duration spaceflight. Bone.

[7] Shinojima A, Kakeya I, Tada S (2018). Association of Space Flight With Problems of the Brain and Eyes. JAMA Ophthalmol.

[8] Rask, J., Vercoutere, W., Navarro, B. J. & Krause, I. *Space Faring: The Radiation Challenge Introduction and Module 1: Radiation Educator Guide*. (National Aeronautics and Space Administration, 2008).

[9] Ohnishi K, Ohnishi T (2004). The biological effects of space radiation during long stays in space. Biol Sci Space.

[10] Hada M, Georgakilas AG (2008). Formation of clustered DNA damage after high-LET irradiation: a review. J Radiat Res.

[11] Murata Y (2015). Histological and Transcriptomic Analysis of Adult Japanese Medaka Sampled Onboard the International Space Station. PLoS One.

[12] Schatten H (1999). Effects of spaceflight conditions on fertilization and embryogenesis in the sea urchin Lytechinus pictus. Cell Biol Int.

[13] Tash JS, Kim S, Schuber M, Seibt D, Kinsey WH (2001). Fertilization of sea urchin eggs and sperm motility are negatively impacted under low hypergravitational forces significant to space flight. Biol Reprod.

[14] Ubbels GA, Berendsen W, Narraway J (1989). Fertilization of frog eggs on a Sounding Rocket in space. Adv Space Res.

[15] Souza KA, Black SD, Wassersug RJ (1995). Amphibian development in the virtual absence of gravity. Proc Natl Acad Sci USA.

[16] Wentworth, B. C. & Wentworth, A. L. Fecundity of Quail in Spacelab Microgravity. *NASA Technical Reports Server* (1996).

[17] Wakayama S (2017). Healthy offspring from freeze-dried mouse spermatozoa held on the International Space Station for 9 months. Proc Natl Acad Sci USA.

[18] Serova LV, Denisova LA, Baikova OV (1989). The effect of microgravity on the reproductive function of male-rats. Physiologist.

[19] Sapp WJ (1990). Effects of spaceflight on the spermatogonial population of rat seminiferous epithelium. FASEB J.

[20] Shiba D (2017). Development of new experimental platform ‘MARS’-Multiple Artificial-gravity Research System-to elucidate the impacts of micro/partial gravity on mice. Sci Rep.

[21] Shimbo M (2016). Ground-based assessment of JAXA mouse habitat cage unit by mouse phenotypic studies. Exp Anim.

[22] Mains, R., Larenas, P. & Hing, A. M. Researcher’s guide to rodent research. *The NASA ISS Program Science Office* (2016).

[23] Andreev-Andrievskiy A (2014). Mice in Bion-M 1 space mission: training and selection. PLoS One.

[24] Ulanova A (2015). Isoform composition and gene expression of thick and thin filament proteins in striated muscles of mice after 30-day space flight. Biomed Res Int.

[25] Tavella S (2012). Bone turnover in wild type and pleiotrophin-transgenic mice housed for three months in the International Space Station (ISS). PLoS One.

[26] Sandona D (2012). Adaptation of mouse skeletal muscle to long-term microgravity in the MDS mission. PLoS One.

[27] Cancedda R (2012). The Mice Drawer System (MDS) experiment and the space endurance record-breaking mice. PLoS One.

[28] Setchell, B. P. & Brooks, D. E. Anatomy, Vasculature, innervation, and fluids of the male reproductive tract in *The Physiology of Reproduction* (eds Knobil, E. & Neill, J. D.) 753–836 (Raven Press, 1988).

[29] Risbridger, G. P. & Taylor, R. A. Physiology of the male accessory sex structures: the prostate gland, seminal vesicles, and bulbourethral glands in *Knobil and Neill’s Physiology of Reproduction* (ed. Neill, J. D.) 1149–1172 (Elsevier, 2006).

[30] Alison MR, Wright NA (1979). Testosterone-induced cell proliferation in the accessory sex glands of mice at various times after castration. Cell Tissue Kinet.

[31] Deaver DR (1992). Effects of caudal elevation on testicular function in rats. Separation of effects on spermatogenesis and steroidogenesis. J Androl.

[32] Meleshko, G. I., Shepelev, Y. Y., Averner, M. M. & Volk, T. Risks to Astronaut Health During Space Travel in *Safe Passage: Astronaut Care for Exploration Missions* (eds Ball, J. R. & Evans, C. H. Jr.) 37–74 (National Academies Press, 2001). 25057582

[33] Ogneva IV (2019). Testes and duct deferens of mice during space flight: cytoskeleton structure, sperm-specific proteins and epigenetic events. Sci Rep.

[34] Singh KP, Kumari R, Dumond JW (2010). Simulated microgravity-induced epigenetic changes in human lymphocytes. J Cell Biochem.

[35] Witze, A. Astronaut twin study hints at stress of space travel. *(Nature news)*, 10.1038/nature.2017.21380 (2017).

[36] Kerr, J. B., Loveland, K. L., O’Bryan, M. K. & de Kretser, D. M. Cytology of the Testis and Intrinsic Control Mechanisms in *Knobil and Neill’s Physiology of Reproduction* (ed. Neill, J. D.) 827–947 (Elsevier, 2006).

[37] R, Bernard, Hinton, T. B. & Orgebin-Crist, M. C. The Epididymis in *Knobil and Neill’s Physiology of Reproduction* (ed. Neill, J. D.) 1071–1148 (Elsevier, 2006).

[38] Takeo T, Nakagata N (2010). Combination medium of cryoprotective agents containing L-glutamine and methyl-{beta}-cyclodextrin in a preincubation medium yields a high fertilization rate for cryopreserved C57BL/6J mouse sperm. Lab Anim.

[39] Toyoda Y, Yokoyama M, Hoshi T (1971). Studies on the fertilization of mouse eggs *in vitro*. The Japanese journal of animal reproduction.

[40] Wakayama S (2009). Detrimental effects of microgravity on mouse preimplantation development *in vitro*. PLoS One.

